# Mental Health Among Elite Youth Athletes: A Narrative Overview to Advance Research and Practice

**DOI:** 10.1177/19417381231219230

**Published:** 2024-01-03

**Authors:** Courtney C. Walton, Rosemary Purcell, Jo L. Henderson, Jeemin Kim, Gretchen Kerr, Joshua Frost, Kate Gwyther, Vita Pilkington, Simon Rice, Katherine A. Tamminen

**Affiliations:** † Centre for Youth Mental Health, University of Melbourne, Melbourne, Australia; ‡ Elite Sports and Mental Health, Orygen, Melbourne, Australia; § Melbourne School of Psychological Sciences, University of Melbourne, Melbourne, Australia; ‖ Centre for Addiction and Mental Health, Toronto, Ontario, Canada; ¶ Department of Psychiatry, University of Toronto, Ontario, Canada; # Faculty of Kinesiology and Physical Education, University of Toronto, Ontario, Canada; ** Department of Kinesiology, Michigan State University, East Lansing, Michigan

**Keywords:** adolescence, child safety, development, ecological models, youth sport

## Abstract

**Context:**

Participation in sports during youth is typically beneficial for mental health. However, it is unclear whether elite sport contexts contribute to greater risk of psychological distress or disorder. The aims of this paper are to highlight conceptual issues that require resolution in future research and practice, and to examine the key factors that may contribute to the mental health of elite youth athletes (EYAs).

**Evidence Acquisition:**

A narrative overview of the literature combined with the clinical and research expertise of the authors.

**Study Design:**

Narrative overview.

**Level of Evidence:**

Level 5.

**Results:**

EYAs experience a range of biopsychosocial developmental changes that interact with mental health in a multitude of ways. In addition, there are various sport-specific factors that contribute to the mental health of EYAs that may become more prominent in elite contexts. These include - but are not limited to - patterns relating to athlete coping and self-relating styles, the nature of peer, parental, and coach relationships, organizational culture and performance pressures, and mental health service provision and accessibility.

**Conclusion:**

A range of critical factors across individual, interpersonal, organizational, and societal domains have been shown to contribute to mental health among EYAs. However, this evidence is limited by heterogeneous samples and varied or imprecise terminology regarding what constitutes “youth” and “elite” in sport. Nevertheless, it is clear that EYAs face a range of risks that warrant careful consideration to progress to best practice principles and recommendations for mental health promotion and intervention in elite youth sport.

**SORT:**

Level C.

Mental health disorders are a leading concern for young people. While pooled prevalence rates have been estimated to be around 13%,^91^ more recent data suggest rates of mental ill-health in youth are growing rapidly.^2^ Further, around half of all mental health disorders emerge before age 18 years, with the midteens representing the peak age of onset.^112^ Young people experiencing mental ill-health may face difficulties across key developmental milestones, including identity and relationship formation, educational and vocational attainment, financial independence, and achieving autonomy.^79^ Despite the clear importance of supporting mental health in youth, evidence suggests that young people report unmet needs and inadequate care.^23^ Better approaches to supporting young people during this developmental period are needed.^81^

Participation in sport is predominantly considered to be supportive to youth mental health.^7,32,69^ Sports participation contributes to broader psychosocial development, by strengthening skills such as communication, responsibility, and emotional regulation.^22,49^ However, many young people drop out of sports during adolescence.^58^ Two major factors associated with dropout are perceptions of insufficient competence and reduced enjoyment of sport.^24^ Potentially, this may speak to concerns regarding the increasingly competitive nature of youth sports.^25^

Not all youth sports environments are equal.^33^ At least by intention, recreational youth sports typically prioritize participation rather than emphasizing a high level of competition and performance.^16^ This participatory approach likely contributes to the many well-established and beneficial outcomes discussed throughout the literature. Young people engaged in highly competitive environments, however, face a range of stressors that may contribute to more challenging experiences. Undeniably, there has been a rapid cultural transition in many countries toward increasingly professionalized youth sports.^25^ These tend to focus on intense and frequent training loads with an emphasis on performance outcomes.^25,58^ Recent trends for “youth-focused” sports in the Olympic Games accentuate this pattern.^6^ For example, the women’s medal winners in street skateboarding in the last Olympic Games at Tokyo 2020 were aged 13, 13, and 16 years. For researchers and practitioners to best support young people in sport, we must think carefully and critically about the nature of these high-performance environments and examine how they interact with young people’s mental health.

The mental health needs of elite and professional adult athletes have received increased research attention in recent years.^92,124^ However, the mental health of youth who participate in “elite” sport contexts (elite youth athletes [EYAs]) has received far less focus.^94^ The aim of this article is to provide an overview of the importance of supporting mental health among EYAs, highlighting inconsistencies that are likely inhibiting consensus on contributing factors. It is important to understand risk and protective factors in this population to better support the development and implementation of appropriate interventions. In this paper, we provide an overview of the literature to explore definitional and conceptual issues and then discuss key considerations from a developmental and ecological perspective.^41^ In doing so, we identify key risk and protective factors, drawing attention to the need for a rigorous and specific research agenda in this population.

## Applying a Developmental Perspective

To recognize the mental health challenges faced by EYAs, it is important to consider the developmental and transitory phases that young people experience more broadly. The mental health needs of EYAs arise in the context of navigating multiple key developmental milestones across diverse biopsychosocial domains. Biologically, from childhood through adolescence and early adulthood, the brain undergoes drastic developmental growth, including changes in myelination, synapse pruning, increasing white matter, and decreasing gray matter.^39,121^ In parallel, young people experience shifts in executive functioning over time, including around risk taking, planning, and capacity to anticipate consequences. In addition, development occurs across intelligence, memory, problem solving, and social cognition.^39^ Adolescence also marks a period where emotional regulation skills tend to mature, which is important given emotional regulation is well established as a protective factor for psychopathology and key target for intervention.^109^

Further significant changes occur as adolescents’ social worlds expand from an initial focus on family, school, and peer relationships, to broader social networks increasingly decoupled from adult supervision and shifting to include intimate relationships.^18^ This is particularly prominent in Western and individualist cultures. Shifts in peer relationships often coincide with changes in family relationships, which can be marked by increased conflict, and both are typically interwoven with expanded identity exploration and autonomy seeking.^78^ Simultaneously, in educational and vocational settings, future planning increases and the impact of academic challenges becoming more pronounced.^31^ Each of these domains undergoes further significant change during the transition to adulthood when adolescent-based extra-curricular activities and services end, and young people typically embark on new educational or vocational endeavors, engage in new relationships, and may establish new living arrangements.

These changing psychosocial environments exert dynamic and bidirectional influences over the course of development,^13^ and, as each of these biopsychosocial developmental tasks is navigated, they may interact with the experiences of EYAs in and outside of sport. Importantly, all of these biopsychosocial changes occur within the adolescent age span, as well as before (late childhood) and after (young adulthood). That is, significant changes occur between the ages of 12 to 13 and 16 to 17. These changes have major implications for a young person’s capacity to manage the various demands and pressures that come with more elite levels of sports competition during adolescence.

The athletic developmental process also occurs alongside these biopsychosocial changes. Several phases of transition occur throughout the pathway of an EYA, such as from participatory to competitive sport, specialization, and entry into adult elite sport.^135,136^ Throughout these phases, certain risk factors and demands become increasingly prominent. For example, performance pressures may increase linearly through higher competitive levels. Others, however, may exist across any competitive stage (eg, peer conflict). The transition into elite sport settings is also a time where EYAs experience difficulties and increased mental health risk.^62^ Further, this transition often occurs at a time where EYAs are experiencing parallel transitions from adolescence to young adulthood, or secondary to higher education, which bring their own accumulating challenges.^136^ It is important to view such transitional periods as long-term processes, rather than singular or isolated events. Notably, sporting organizations that understand the demands, resources, and barriers to effective transitions have shown better player development outcomes.^83^ Thus, advancing research in the area of mental health among EYAs requires a developmental perspective that considers the multiple developmental milestones that youth are navigating alongside their sport participation.

## Establishing Conceptual Clarity: who are EYAs?

Before we examine factors that may influence mental health among EYAs, it is necessary to first consider the terminology regarding both “youth” and “elite.” Our observation is that inconsistent language is prominent, and highly heterogeneous samples are frequently combined in research on youth sport. In an example of how this cohort has been described, a 2008 International Olympic Committee statement suggests “the elite child athlete is one who has superior athletic talent, undergoes specialized training, receives expert coaching and is exposed to early competition.”^85^ Despite this proposed definition, conceptual clarity and consistency regarding both terms (“youth,” “elite”) is variable, and thus needed. We recommend the term EYAs to describe this population, as the term emphasizes a type of “youth athlete,” rather than simply a younger “elite athlete”; the latter reinforcing some of the problematic win-at-all-costs perspectives seen in elite sport.

Continuing debate exists as to the appropriate age brackets that define periods of youth and adolescence.^102^ Categories defined as “childhood,” “adolescence,” and “young adulthood” as well as “youth” or “young people” are commonly used in unclear or overlapping ways, and frequently with varying levels of consistency. Considering these varied categories, youth is often considered to span approximately 10 to 25 years of age. Indeed, a recent review explicitly characterized EYAs as those aged 10 to 24 years.^26^ However, using such a broad definition has limited value in a sports context, where both environments and developmental stages vary so significantly across this period. To illustrate, consider the environment that a 12-year-old academy athlete finds themselves in, compared with a 20-year-old in a top professional league. Of course these are incomparable, yet researchers have frequently combined these varying presentations under the banner of youth. Our preliminary scan of the literature uncovered broad age ranges across studies explicitly describing “EYAs” with participants ≤12 or ≥20 years old.^53,101,138^

There are issues at both the upper and lower bounds of this age range (10-25 years) classified as youth, when it comes to meaningful translation into competitive sport. At the younger boundary, we must seriously consider how early is too early to be considered as “elite,”^57^ particularly given that early specialization is not linked with additional performance benefits and may in fact lead to worse psychosocial and injury-related outcomes.^3,9,59^ At these earlier ages, the potential for chronic and long-term injury and physical harm is increased due to limited physical growth and maturation.^5^ Acknowledging that important age norms vary across sports (eg, gymnastics as compared with rugby union),^55^ we advocate that sport participation during childhood (ie, <13 years of age) should be primarily for developing opportunities for exploration and playing of diverse sports,^21^ and align with the Developmental Model of Sport Participation whereby youth athletes pursuing elite pathways do so after approximately 13 years of age.^20,21^

At the upper boundary, concerns arise when we consider that transitions into professionalized contexts often occur much earlier than age 25 years. Indeed, youth in many sports (eg, various football codes around the world) transition to professional adult sport around age 18 years, at which point the athletes’ psychosocial environment changes drastically, compared with a more youth-focused developmental or academy environment. Even where professionalization may not yet have occurred, the common cessation of secondary schooling around age 18 years and initiation of postsecondary activities bring new and more intensive training practices (eg, National Collegiate Athletic Association sports in the United States). While acknowledging many elite sports include underage categories (eg, Under 21s and Under 23s), athletes in these categories are nevertheless at a significantly different athletic and developmental stage and engage with a more mature sports environment by way of facilities available to them, living arrangements, financial autonomy, sporting expectations, and personal responsibilities. This is supported by the fact that athletes over the age of 18 years are ineligible to compete at the Youth Olympic Games, where athletes must currently be aged between (and including) 15 and 18 years.

Thus, we suggest that the term EYA be restricted to a more constrained age range, and propose ≥12 to ≤17 years old as a practical categorization. Children under the age of 12 years should not be pressured through traditional “elite” sports environments given the risks to health and limited biopsychosocial development at this age. On the other hand, age 18 years aligns with many well-established social milestones that lead into early adulthood. Therefore, this age range generally corresponds with adolescence and secondary schooling, and aligns with our argument that the key element here is participation in developmental or pathway contexts before adult elite or professional sport.

Just as murky as the term “youth,” is that of “elite.” Our exploratory search of the literature found variable criteria on EYA classification. Two major attempts have been made to provide a taxonomy for eliteness in sports research reporting^80,115^; however, neither considers youth in a meaningful way. In their Participant Classification Framework, McKay et al^80^ propose that future iterations are needed to consider the introduction of age categories, which we certainly support. We suggest that in youth sports, “elite” be used to specify the context in which a young person trains and competes, rather than their performance. That is, we propose that it is engagement with these cultures and contexts that is most important to account for when considering mental health in youth, rather than previous taxonomies that focus on the athlete’s ability and success (eg, quantifying “success at the highest level” or their “Olympic or World medals”).^80,115^ This is aligned with the aforementioned definition from Mountjoy et al,^85^ who describe environmental factors of specialized training, expert coaching, and early competition. Acknowledging that a more comprehensive analysis to accurately classify elite in youth is necessary but beyond the scope of our review, we broadly separate elite contexts from recreational or competitive youth sports where the following 3 criteria are met:

1. A primary focus on performance outcomes, rather than broader goals of psychosocial development, enjoyment, and participation.
2. A priority placed on involvement in sports at the expense of other critical psychosocial and educational experiences and nonsports relationships.
3. An explicit or implicit underlying goal of progression to adult elite, collegiate, or professional sports.

## Conceptualizing the Mental Health of EYAs Through Ecological Systems

Common approaches to understanding youth and elite sports environments include Systems Theory,^10,27,123,127^ and variations of Bronfenbrenner’s Ecological Model.^11,12,21,38,123^ Any consideration of how best to support the mental health of EYAs must consider the broader context surrounding the EYA, and thus these approaches provide a valuable lens through which to consider elite youth contexts. Thus, ecological models are critical for understanding how a person’s mental health is affected both positively and negatively by the various interrelated systems in which they participate.^93,95^ Using this lens to consider elite youth sport, we first outline “Individual Factors” that contribute to mental health. We then cover “Interpersonal Factors,” including relationships with those who have direct and frequent contact with the EYA (eg, coaches, teammates, and parents). Further out are “Organizational Factors,” which include aspects of the wider sporting or educational environment. Finally, we consider “Societal Factors,” which relate to those aspects of broader society that may impact the EYA, including media and social media, community awareness, and stigma. In considering each of these factors, it is also important to acknowledge in parallel the effect of developmental functioning. Thus, the relationship between an EYA and their coach and parents changes significantly from early through to late adolescence by way of their psychological growth and developing autonomy. In the following section, we summarize key factors in each of these systems that are interrelated and may contribute in both positive and negative ways to the mental health of EYAs.

## Key Factors Contributing to the Mental Health of EYAs

### Individual Factors

A range of individual factors undoubtedly contribute in protective or risk-enhancing ways to mental health. The extant literature has examined primarily how these individual factors impact risk for burnout, disengagement, and performance anxiety for EYAs, with few studies exploring risk for specific mental health outcomes. Individual factors that impact these potential precursors to mental ill-health include self-imposed performance pressures, maladaptive perfectionism, and overtraining.^46,51,111,114^ Beyond these sport-specific factors, general risk factors include avoidance-focused coping styles, low mental health literacy, exposure to adverse life events, and poor sleep.^42,43,72^ Most of these factors are not experienced exclusively by EYAs, and many continue to impact adult athletes.^63^ However, they may interact with different periods of developmental stages in more complex ways during youth. It is also important to understand how these factors change over time. For instance, maladaptive perfectionism has been found to be higher in EYAs than in adult elite athletes.^51^

Aspects of an athlete’s identity, including, but not limited to, gender, sexuality, race and ethnicity, Indigeneity, ability/disability, and the intersection of these identities, can also influence experiences of mental health. For example, eating disorders have been shown to be far more prevalent among female as opposed to male EYAs.^74^ While limited research exists among EYAs, it is well established that elite athletes as well as those in the broader community who identify as female, sexually- or gender-diverse, of a racial minority, and those with a disability often report more experiences of sexual, psychological, and physical harms.^44,126^ In addition, young people of any gender - but especially boys - who strive to maintain orthodox masculine ideals may demonstrate behaviors that negatively effect their mental health, such as reduced help-seeking or the suppression of pain leading to further injury.^104,116^ These tendencies align with a perceived need to uphold hegemonic forms of masculinity by trying to minimize association with perceived weakness or vulnerability, and follow cultural scripts that emphasize toughness and control.^98,107^ In sum, there are a number of individual factors that may intersect with interpersonal, organizational, and societal factors to impact the mental health of EYAs. However, research on these factors is limited with EYA populations specifically, and more information is needed to advance knowledge in this area.

### Interpersonal Factors

There is a plethora of evidence demonstrating the importance of interpersonal factors and social supports on the mental health of youth,^100^ although less is known specifically in the EYA population. Nonetheless, it is clear that EYAs interact with, and are influenced by, several key social agents including parents, coaches, peers, and teammates, as well as their social media environment; all of which can influence mental health.

#### Parental Influences

Despite a lack of research specifically examining the influence of parents on the mental health of EYAs, research in recreational and competitive sport settings provides insight on the positive and negative influences that parents can have on the experiences of EYAs. Parents typically facilitate their child’s sport participation and support them through sustained involvement in elite sport.^4^ Given the investment of time, money, and emotional support that parents provide for EYAs,^60,61,67^ parents are generally viewed as playing a substantial and positive role in their child’s sport experiences. Indeed, EYAs’ perceptions of their parents’ reinforcement and encouragement are significantly associated with their intrinsic motivation, self-efficacy, psychological skills, and performance in sport.^119^

However, EYAs may also experience excessive parental pressure related to sport, along with an overemphasis on talent development and criticism about their performance.^64,117^ Negative parental behaviors such as exhibiting exceedingly high expectations, an overemphasis on winning, and inappropriate pressure to perform have been linked to increased athlete anxiety, decreased perceptions of competence, and decreased sport enjoyment.^47^ Parental pressure has been linked to youth athletes’ perfectionistic strivings and perfectionistic concerns,^71^ and parental climates that emphasize concerns about failure are associated with perfectionism and burnout among competitive junior athletes.^45^ In addition, more positive parent-child relationships play a protective role against the formation of maladaptive eating attitudes such as severe food restriction,^106^ and critical parental comments about an EYA’s body or eating behaviors have been associated with disordered eating and body image dissatisfaction in aesthetic sports.^37^ Furthermore, parent communication, concerns, and practices-related to their child athlete’s weight and shape are associated with their child’s compulsive exercise and disordered eating behaviors.^70^

It is important to consider that, depending on developmental functioning and local policies around health care, EYAs are often dependent on their parents/legal guardians for accessing professional mental health care.^68^ Parents may be among the first people in the EYA’s microsystem to notice changes in mood or behaviors that could indicate mental health concerns. Indeed, adolescent male athletes have reported that parents are well-positioned to notice their mental health difficulties.^116^ Here, parental mental health literacy and attitudes toward mental health are important factors that may influence the detection of concerns and the use of health services, as parents who are better able to perceive mental health problems among their children are more likely to seek support.^68,118^ In addition, parental mental health may also influence EYAs’ access to mental health care, as parents who have experienced psychiatric disorders may be more likely to notice and report mental health concerns among their children.^118,139^ Although research in this area is nascent, mental health literacy interventions for EYAs and their parents show promise for improving mental health and well-being in this population.^26^

#### Peer Influences

Adolescence marks a developmentally critical period in which athletes are highly sensitive to influence from peers and teammates. Although research specifically focusing on EYAs is rare, there is evidence suggesting that peers and teammates may significantly influence the mental health of EYAs in both positive and negative ways. Adolescent athletes who participate in team sports tend to report fewer depressive symptoms compared with those who participate in individual sports,^90^ and there is evidence that social support from teammates is positively associated with psychological well-being and negatively associated with burnout among adolescent athletes.^17,88^ Indeed, positive teammate dynamics and peer acceptance contribute to greater enjoyment of sport and greater self-worth, and peer motivational climate is an important predictor of youth athlete burnout.^110^

Whereas there are numerous positive interpersonal effects from peers, EYAs may also experience harmful peer interactions such as bullying and victimization.^86^ Sport environments are known to normalize practices such as hazing, which involves team members forcing incoming members to participate in humiliating and often dangerous behaviors to be accepted by the group.^110^ In a large study of over 11,000 college athletes, 47% reported experiencing hazing during high school, suggesting such practices are common in youth.^1^ Such forms of interpersonal violence from peers are key risk factors for mental ill-health, and remain pervasive in competitive sport environments.^110^ Further, the prevalence and effects of harmful interpersonal interactions may be exacerbated in contemporary youth contexts due to electronic communication and social media. Though research on social media in youth sport contexts is nascent, there is evidence from nonsport research that social media can negatively impact adolescents’ mental health, with cyberbullying playing a relevant predictive role.^34,56^ As EYA interactions with peers and teammates extend beyond the sport environment through digital spaces,^30^ there is a need for additional research in this area.^36^

Peer influence is also known to relate to potentially risky substance use. Though rates among youth are mixed and depend on the specific substance,^96,120^ peer and teammate influences are cited as a key reason for an athletes’ substance use. For instance, in a study of EYAs in Norway, participation in team sports was associated with higher use of snus (a tobacco product), which the authors attributed to peer pressure.^75^ Similarly, others have found that high school students who participated in team sports reported greater alcohol use, and suggested that belonging to teams that have normalized drinking may contribute to increased alcohol use.^120^

An important positive effect of peer and teammate influence on mental health is that, similar to parents, peers may be well placed to detect mental health problems. For instance, EYAs have reported that persons who experience eating disorders may not acknowledge their own maladaptive eating patterns, and that others may be in a better position to recognize the symptoms of disordered eating and encourage help-seeking.^43^ Indeed, Gulliver et al^43^ found that positive attitudes toward help-seeking from teammates (along with coaches, parents, and other key social agents) were considered a facilitator of help-seeking for mental ill-health. In addition, the documented positive effects of social support from teammates suggest that teammates could be a key source from which EYAs can seek help for psychological distress.^88^

#### Coaching Influences

Research examining the influence of coaches on the mental health of EYAs is similarly limited, despite the fact that approaches to coaching likely differ significantly in elite youth sport contexts as compared with recreational youth sports. When coaches misuse their positions of power and authority, EYAs may be more prone to mental health challenges. For example, athletes who have experienced maltreatment, including neglect, and sexual, physical, and psychological abuse by their coaches cite more pervasive mental health consequences, including depression and anxiety, eating disorders, and post-traumatic stress disorder.^54,87,113,126^

As primary leaders in youth sports environments, coaches are also well placed to identify, address, and support a number of mental health needs in young people.^76^ Coaches often therefore act as potential gate-openers for mental health support among adolescent athletes.^8,14,76^ As noted earlier, Gulliver et al^43^ found that EYAs perceived a coach’s positive attitude toward mental health help-seeking as a decisive facilitator in accessing support. Conversely, other participants in this study were reluctant to seek help due to fears about the negative judgments and implications potentially imposed by their coach. This barrier to help-seeking is influenced by the strength of the coach-athlete relationship, which has been shown to shape the extent to which a youth athlete may feel comfortable in reaching out to their coach for help.^14^ Therefore, while coaches have expressed the challenges of balancing performance and mental health,^65,76^ they must be supported to nurture cultures that prioritize and promote mental health awareness and positive interpersonal dynamics.

In non-elite youth settings, many coaches have acknowledged their responsibilities include helping to identify and refer to appropriate supports if an athlete exhibits symptoms of mental ill-health.^35^ However, this relies on coaches possessing appropriate mental health literacy and the skills to accurately detect warning signs among EYAs, as well as sufficient referral networks, supports, and available resources. This is supported by evidence that mental health literacy is associated significantly with early intervention, prevention, and promotion among youth coaches.^29^ It is therefore postulated that coaches need the appropriate knowledge, competence, and beliefs to effectively monitor and support the mental health of EYAs.^29^

### Organizational Factors

The broader context in which EYAs train and compete has important implications for their mental health and help-seeking. Elite sport contexts, particularly for youth, have been criticized for a win-at-all-costs approach that prioritizes performance outcomes over the holistic health and well-being of EYAs. For example, a recent position paper by the Centre for Sport and Human Rights has equated elite sport for youth as a form of child labor on the basis that EYAs can engage in many hours of training per week that confer financial and professional benefits for adults in the youth’s circle.^16^ Together, early specialization, intensive commitment to the singular pursuit of sport, increasingly strict training and lifestyle demands, and a win-at-all-costs approach contribute to mental health challenges often seen during the retirement transition.^103,137^

Organizations can influence mental health through the leadership and cultures that are maintained and promoted. For example, at their worst, disturbing high profile cases have demonstrated how toxic, secretive, and abusive practices from those in positions of power and leadership have been tolerated in pursuit of sports achievement.^84^ On the other hand, elite youth sports organizations that promote aspects of psychological safety (ie, open and nonjudgmental communications) and reduce risks of harm are likely more supportive of mental health.^130^ Although this is poorly understood in the context of elite youth sport due to limited research, recent evidence suggests that elite athletes who perceive their organizational climate to be supportive of mental health demonstrate reduced rates of psychological distress.^99^

In addition to setting cultural norms and standards, organizations also play a critical role in ensuring that sufficient policies and protocols are in place to support the mental health of young people. While all youth sports organizations have a duty of care to support both the physical and mental health of their members, elite contexts are typically privileged to have more resources and financial capabilities, which opens up both opportunity and responsibility. For example, elite youth sports organizations may be well placed to ensure EYAs have access to mental health professionals associated with the organization, either internally or through clear referral pathways. When the organization is able to take a more complete approach to supporting mental health, better outcomes can be expected. For example, a comprehensive intervention that took place over a year in Norwegian elite sports schools showed evidence of preventing the emergence of eating disorders in female EYAs.^73^

The role of elite sports organizations in mental health service provision varies significantly by context,^82^ and we are not aware of published processes specific to elite youth sports. However, a range of examples exist in elite sport that can potentially be used as a guide for implementation in youth sports. For example, a recent evidence-informed framework provides guiding principles and actionable recommendations for promoting mental health in elite sports, leaning on published literature and expertise from various stakeholders across sports medicine and clinical sport psychology.^95^ There are also examples in Canada and Australia of how elite sports organizations and systems can set out accessible procedures for athletes to receive mental health treatment and support.^97,122^

At the very least, organizations should ensure training in mental health literacy and promotion for young people,^66^ their parents,^50^ and coaches or other staff working in these environments.^28^ Research suggests that, generally, stakeholders in youth sports are willing to participate in these types of initiatives.^89^ This type of training ensures that all those who come in contact with EYAs are sufficiently skilled in recognizing and responding to potential distress, to ensure that young people are supported in accessing care where needed. Organizations can also utilize readily available mental health toolkits and guidelines to ensure they are enacting best practice and evidence-informed principles for mental health promotion.^128^ An example of a recent and well-developed intervention is “Ahead of the Game” - a multicomponent intervention delivered via sports clubs that has demonstrated a range of positive outcomes related to mental health.^125^ Though this intervention is not targeted specifically at elite youth sport and has been tested for efficacy primarily among boys, it is an example of how structured interventions can be implemented in sports by an organization to increase mental health literacy. However, sports organizations cannot simply rely on brief interventions and must instead ensure they implement comprehensive, open, and accessible structural supports and safety nets.

Overall, organizations can support mental health of EYAs by removal of physical and psychological harms, promoting positive cultural norms, and ensuring access to appropriate care. Elite youth sports organizations must be able to shift from a performance-only focus, toward more developmentally informed approaches that support mental health. In addition, they can assist or even mandate that all persons in these environments be upskilled on recognizing and responding most effectively to signs of potential mental ill-health.

### Societal Factors

Finally, at the level of community and society, young people face many barriers to mental health services. Indeed, the majority of youth outside of elite sports contexts who are living with significant mental health concerns do not receive specialized mental-health-related treatment.^19,40^ Barriers to service engagement for youth include lack of awareness, stigma, system fragmentation, long wait-times, and lack of developmentally appropriate services.^48,108,134^ Access of EYAs to effective mental health and/or substance use services may be further impacted by limitations in clinicians’ familiarity with the unique strengths, challenges, pressures, and contexts that EYAs experience. Elite adult athletes place a particular value on their treating clinicians being able to recognize the specific demands of competitive sport culture.^52^ Thus, increasing knowledge about the challenges faced by EYAs, and upskilling the relevant mental health practitioners working with the unique needs of this population, is an important goal.^133^

Societal attitudes about mental health in sport can also influence EYA access to mental health care. There is a common misconception that athletes do not experience mental health concerns given their outwardly “fit” appearance and “healthy” lifestyle behaviors such as exercise and regimented nutritional intake. This is particularly relevant to the identification of EYAs at risk or already experiencing mental health concerns, as persons working with athletes may sidestep exploration of psychological health in favor of physical explanations,^105^ or consider symptoms as simply reflective of overtraining or a “normal” response to their athletic pursuits. Although there has historically been high levels of societal stigma around mental health disorders, a cultural change is apparent. Discourse around mental health among elite athletes is becoming increasingly common more recently, with well-known athletes speaking openly about their challenges with mental ill-health. It is likely that this discourse playing out in broader society will gradually influence the readiness of EYAs and young people in the community more broadly to engage with mental health support.^131^ Indeed, Swann et al^116^ found that male athletes reported that they would be more engaged in mental health discussions when associated with a sporting role model.

## Call to Action: a Research Agenda to Guide Best Practice for Mental Health OF EYAs

While a focus on mental health in the contexts of elite/professional adult or recreational youth sport has increased dramatically in the last decade,^92^ EYAs have been left behind.^94^ This is particularly concerning given that EYAs may be at high risk, balancing critical developmental milestones alongside the pressurized sport-specific factors outlined earlier. To ensure that the mental health of young people in elite sport is supported, we call on more research exploring mental health among EYAs. In particular, we propose that the current literature suffers from inconsistent conceptualization or separation of elite youth sport, leaving conclusions about this population disorganized. While the development of a comprehensive youth-specific framework of elite sport is necessary,^80,115^ here we have defined EYAs as 12 to 17-year-olds engaged in sport that is primarily performance-focused, often at the expense of critical psychosocial and educational experiences, and with a goal of progression to elite adult sport. Researchers should aim to be specific about describing the competitive contexts they are studying, and literature reviews of youth sport should consider inclusion criteria that focus explicitly on either elite or recreational contexts, given the clear differences that exist.

In expanding the literature base, youth-engaged research,^77^ which centers the lived experience of EYAs throughout the process, will be critical to developing acceptable and feasible interventions as well as best-practice organizational processes. This research should prioritize capturing accurate prevalence rates of mental health disorders in elite youth sport, and utilize rigorous methodologies such as comprehensive longitudinal and cohort data to understand risk and protective factors based on the ecological lens described earlier. Such work is difficult and time-consuming to conduct, and thus targeted funding opportunities from leading sports organizations and funding bodies are necessary to make this research possible. Given previously described intersections between individual factors, such as gender or racial identity and mental health, it is critical that this research pays ample attention to considerations around equity, diversity, and inclusion. For example, a recent systematic examination of participants in sport psychology research found that studies with elite athlete samples drastically underrepresent women and girls.^129^ Given gender differences reveal typically higher mental ill-health for female-identifying elite atheltes,^132^ and adolescents more broadly,^15^ this should be addressed thoughtfully in future research with EYAs.

## Limitations

The primary limitation of this paper is the absence of a systematic search or preregistered protocol. Instead, we chose to employ an overview and narrative synthesis of selected literature,^41^ which was deemed more appropriate due to the paper’s broader aims. We also did not complete a quality appraisal of the included studies, and we were therefore unable to assess the strength of the evidence in the included papers. The review process did not involve stakeholder engagement, which would be valuable in future work to support effective research impact and knowledge translation of research results. A fundamental limitation of the extant literature included in this paper is that there is poor conceptual clarity along with heterogeneous interpretations as to what constitutes an EYA among studies of young people engaged in sport. Consequently, making dependable conclusions about the specific factors contributing to mental health in this population is extremely challenging.

## Conclusion

Elite youth sports environments have the capacity to support youth athletes’ mental health. Alternatively, they may put certain young people at risk for mental ill-health, or accentuate pre-existing conditions. Unfortunately, the field is limited by imprecise understandings of EYAs’ mental health, delaying best practice principles for early intervention and treatment. We call on researchers to think critically and carefully about how best to categorize and serve this population, implementing rigorous research methodologies to uncover what can be done to best support the mental health of young people in elite sport.
